# Can type 1 diabetes be prevented or reversed?

**DOI:** 10.1111/1753-0407.13572

**Published:** 2024-05-13

**Authors:** Zachary Bloomgarden

**Affiliations:** ^1^ Department of Medicine, Division of Endocrinology, Diabetes and Bone Disease Icahn School of Medicine at Mount Sinai New York New York USA

The worldwide annual incidence of type 1 diabetes (T1D) among children and adolescents is approximately 200 000 persons, half age ≤15,^1^ with an additional 330 000 over age 20 developing T1D annually. The worldwide prevalence of T1D 8 760 000, more than 7 000 000 of whom are over age 20.^2^ We now recognize that there are a substantial number of persons without overt diabetes who may nevertheless be considered to have T1D: those with normal blood glucose but evidence of an autoimmune response to islet autoantigens, considered to have stage 1 T1D; those with abnormal blood glucose in the pre‐diabetes range as well as islet autoantibodies, considered to have stage 2; those with clinical diabetes but still evidence of β‐cell reserve, Stage 3; and those with established T1D and progressively declining β‐cell reserve, stage 4.^3^ Although we know of the existence of stages 1 and 2, it has not been easy to determine their prevalence. In a study of 153 854 youths age 2–10 in Bavaria, Germany, 153 313 screened negative for islet antibodies, of whom 18 developed T1D, 541 screened positive, of whom 447 had multiple autoantibodies; 293 were in stage 1, 61 of whom progressed to Stage 3, 30 were in stage 2, of whom 16 progressed to Stage 3, 14 were Stage 3, 83 declined staging, of whom 10 progressed to Stage 3. Thus, 0.35% of the screened children were antibody‐positive, of whom 101 (less than 20%) developed T1D during the 2‐3 year period of observation.^4^ As the short‐term disease progression risk is low, prolonged observation of a relatively large number of persons from a relatively rare population will be required to fully characterize stages 1 and 2 of T1D; further, the approaches used in assessing potential treatment approaches are hampered by the poor reproducibility of the oral glucose tolerance test, by the effect of variable degrees of insulin sensitivity on insulin secretion and hence on C‐peptide levels, and by the benefit of continuous glucose monitoring‐guided closed‐loop insulin pump systems in controlling glycosylated hemoglobin (HbA1c) and in minimizing hypoglycemia.^5^

Most studies of the pathogenesis of T1D suggest that β‐cell destruction is mediated by T lymphocytes, with the role of islet cell antibodies in the process uncertain. For those not having diabetes, the effects of autoreactive effector T cells are counterbalanced by regulatory T cells, only with those having an expanded or resistant population of effector T cells or impaired regulatory T cells going on to develop T1D.^6^

A number of randomized controlled trials have been carried out to assess potential approaches to T1D prevention in stages 1 and 2; one of two diet‐based interventions with avoidance of cow's milk suggested benefit, one trial was negative with avoidance of gluten exposure, two were negative with administration of nicotinamide, and four were negative with administration of insulin orally or parenterally.^7^ Teplizumab is an antibody to CD3, a protein comprising part of the T cell receptor complex recognizing the major histocompatibility complex (MHC) glycoprotein gene products that present peptides to the T cell receptor of antigen‐presenting cells. In a study of 76 persons aged 8–49 with ≥2 positive antibodies and fasting glucose 100–125 or 2‐h glucose 140–200, a 14‐day teplizumab infusion resulted in 45% of treated persons, but 73% of those receiving placebo, developing diabetes, with median time to diagnosis 50 versus 25 months. Adverse effects included lymphopenia, rash, leukopenia, anemia, low IgM, thrombocytopenia, and liver function abnormality, with serious infection developing in 9% of treated persons but none of the controls, and one case of cytokine release syndrome.^8^

There is more information about immunomodulatory approaches to established T1D (Stage 3). A study of teplizumab administration in two 12‐day infusions, 26 weeks apart, to 217 persons aged 8–17 with ≥1 positive autoantibody, T1D developing within 6 weeks, and peak‐stimulated C‐peptide ≥0.2 was positive for its primary endpoint of preservation of C‐peptide, in 95% of those receiving immunotherapy versus 78% of 111 persons receiving placebo; no difference in A1c, time in range, or hypoglycemia was reported, and there was equivocal difference in insulin dose. Adverse effects included headache, gastrointestinal symptoms, abnormal liver function, rash, lymphopenia, and mild cytokine release syndrome.^9^ A meta‐analysis of multiple 1‐ and 2‐year studies of teplizumab and the related agent otelixizumab confirmed benefit in C‐peptide preservation, and lack of significant difference in HbA1c levels.^10^

Janus kinase (JAK) inhibitors act at the adenosine triphosphate binding site of these enzymes, suppressing a number of signaling pathways leading to immunomodulatory effects that have shown benefit in malignancies, inflammatory diseases such as rheumatoid arthritis, and graft versus host disease^11^; a study of the JAK inhibitor baricitinib administered to 60 persons with T1D onset within 100 days and peak‐stimulated C‐peptide ≥0.2 found preservation of C‐peptide in 95% versus 79% of 30 persons receiving placebo, with reduction in insulin requirement and lower HbA1c (albeit from a lower baseline).^12^ Again, this is not an altogether benign treatment, with the product information citing potential adverse effects including “risk of serious bacterial, fungal, viral and opportunistic infections leading to hospitalization or death… Monitor all patients for active TB during treatment, even patients with initial negative, latent TB test… Higher rate of all‐cause mortality… Malignancies… MACE… [and] Thrombosis.”^13^ Rituximab, a monoclonal antibody to CD20, a protein present on B‐lymphocytes, was administered to 81 persons with T1D onset within 100 days, positive anti‐islet antibodies, and peak‐stimulated C‐peptide ≥0.2, showing lower HbA1c and insulin dose requirement over 30 months.^14^ Again, a number of potential side effects are cited in a “black box” in the product information, “fatal infusion‐related reactions, severe mucocutaneous reactions, hepatitis B virus reactivation and progressive multifocal leukoencephalopathy.”^15^ A comparison of multiple potential agents suggested that the greatest degree of C‐peptide preservation was seen with teplizumab and anti‐thymocyte globulin, with lesser effect seen with alefacept, a fusion protein that impairs CD2‐mediated costimulation and specifically targets memory T cells, abatacept, a fusion protein that impairs co‐stimulation needed for full T lymphocyte activation, and rituximab.^16^

Potentially less toxic approaches are being considered. Antigen‐specific epitope‐based immunotherapy may lead to restoration of antigen‐specific tolerance.^17^, ^18^ Immune checkpoint modulators,^19^ chimeric antigen receptor‐based cell therapy,^20^ and other novel approaches to disease‐modifying immunotherapy^21^ are all being studied and some of these may prove safe and efficacious. In our earlier commentary on the use of pro‐proliferative agents for early T1D treatment we noted a preliminary study with the calcium channel‐blocking agent verapamil having the potential to inhibit thioredoxin‐interacting protein, reducing β‐cell glucotoxicity and apoptosis.^22^ A subsequent controlled trial randomized 88 children and adolescents aged 7–17 with newly diagnosed T1D to verapamil versus placebo; mean C‐peptide was 30% higher at 52 weeks with verapamil, with 95% of treated patients versus 71% of those receiving placebo having stimulated C‐peptide ≥0.2 pmol/mL.^23^ This approach seems for now to be safer and equally efficacious to that with immunomodulatory agents, and it is noteworthy that the cost of two 12‐vial courses of Tzield (teplizumab) would be $332 400, while that of a 30‐day supply of verapamil in the United States ranges from US$5–40, for a yearly cost between US$60–480.
